# Understanding the legal trade of cattle and camels and the derived risk of Rift Valley Fever introduction into and transmission within Egypt

**DOI:** 10.1371/journal.pntd.0006143

**Published:** 2018-01-19

**Authors:** Sebastian Napp, Veronique Chevalier, Núria Busquets, Paolo Calistri, Jordi Casal, Mohamed Attia, Rehab Elbassal, Heba Hosni, Hatem Farrag, Noura Hassan, Rasha Tawfik, Sohair Abd Elkader, Shahin Bayomy

**Affiliations:** 1 CReSA-IRTA, Barcelona, Spain; 2 CIRAD, UPR AGIRs, Montpellier, France; 3 Food and Agriculture Organization of the United Nations (FAO), Rome, Italy; 4 IZSAM, Teramo, Italy; 5 UAB, Barcelona, Spain; 6 Central Administration of Preventive Medicine. Ministry of Agriculture and Land Reclamation, Cairo, Egypt; 7 Department of Epidemiology. Ministry of Agriculture and Land Reclamation, Cairo, Egypt; 8 Quarantine & Inspection Department. Ministry of Agriculture and Land Reclamation, Cairo, Egypt; Princeton University, UNITED STATES

## Abstract

Rift Valley Fever (RVF) is a mosquito-borne zoonosis, which may cause significant losses for the livestock sector and have serious public health implications. Egypt has been repeatedly affected by RVF epidemics, mainly associated to the importation of animals from sub-Saharan countries, where the disease is endemic. The objective of our study was the improvement of the surveillance and control strategies implemented in Egypt. In order to do that, first we evaluated the legal trade of live animals into and within Egypt. Then, we assessed the risk of Rift Valley Fever virus (RVFV) transmission within the country using a multi-criteria evaluation approach. Finally, we combined the animal trade and the risk of RVFV transmission data to identify those areas and periods in which the introduction of RVFV is more likely. Our results indicate that the main risk of RVFV introduction is posed by the continuous flow of large number of camels coming from Sudan. The risk of RVFV transmission by vectors is restricted to the areas surrounding the Nile river, and does not vary significantly throughout the year. Imported camels are taken to quarantines, where the risk of RVFV transmission by vectors is generally low. Then, they are taken to animal markets or slaughterhouses, many located in populated areas, where the risk of RVFV transmission to animals or humans is much higher. The measures currently implemented (quarantines, vaccination or testing) seem to have a limited effect in reducing the risk of RVFV introduction, and therefore other (risk-based) surveillance strategies are proposed.

## Author summary

Rift Valley Fever (RVF) is a mosquito-transmitted disease, which may affect animals (ruminants and camels) and cause severe disease. Egypt has been affected by RVF in the past, and it is considered at risk because of the importation of animals from endemic countries. We developed a map of the risk of RVFV transmission by vectors using current knowledge on the disease, and then combined it with animal trade data to identify the areas at the highest risk for RVFV introduction. Our results indicate that the main risk is posed by camels imported from Sudan, when they are transported to animal markets or slaughterhouses, which are generally located in areas where the risk of RVFV transmission is much higher. In fact many of them are located in the proximities or within the main cities, resulting in an increased risk of RVFV transmission to humans.

## Introduction

Rift Valley Fever virus (RVFV) is an arbovirus which belongs to the *Phlebovirus* genus, within the family *Phenuiviridae* (order *Bunyavirales*). It is the causative agent of Rift Valley Fever (RVF) that may affect both humans and animals, mainly ruminants, but also camels. In livestock RVF causes large number of abortions (abortion storms) and high mortalities in young animals. In humans, RVFV infection generally causes a self-limiting, acute and febrile illness, although in some cases disease progresses to more severe forms, with neurological disorders, blindness, hemorrhagic fever or thrombosis [1]. RVFV infection of animals may occur by the bite of an infected mosquito (mainly of the *Culex* or *Aedes* genera) or through direct contact with infected animal tissues or fluids [2]. Infection via mosquito bites is considered the primary mode of transmission in the first stages of the epidemic, while contact with contaminated material is more relevant during the amplification stage. RVFV within infected tissues is quite resistant to inactivation and may remain infectious for a few days [2]. In fact, the majority of human cases are likely to occur in farmers, veterinarians or slaughterhouse employees due to contact with infected material [3].

RVF may have a dramatic consequence on producers and livestock industries, including the impact on international trade [4]. The public health impact of RVF may also be severe, as demonstrated by the 200,000 people infected and 600 fatal cases reported in the 1977 epidemic in Egypt [5]. Besides that epidemic, Egypt has been affected by RVF in 1993, 1994, 1997, and finally in 2003 [6]. In fact, Mroz and collaborators [7] have recently reported the low level circulation of RVFV in some areas of Egypt.

The continuous importation of viraemic ruminants or camels, mainly from Sudan, was considered the main source of introduction of RVF into Egypt [6]. Furthermore, those importations may also result in the introduction of other exotic diseases such as the Middle East Respiratory Syndrome coronavirus (MERS-CoV) [8]. RVF is considered to be endemic in sub-Saharan African countries (including Sudan), with major outbreaks associated with periods of heavy rainfall and flooding [9]. Socio-economic changes, with human population growth and the associated increased demand for meat, or changes in the meat market prices could result in greater controlled and uncontrolled transboundary movements of livestock. A consequence of all those changes would be an increase of the risk of introduction of RVFV into areas of the Mediterranean basin and the Middle East [3]. While surveillance and diagnostic methods for RVFV are available, RVFV control is hindered by the difficulties of vector control, and the fact that vaccines are only available for ruminants, with either inactivated vaccines (with a limited efficacy) or live attenuated vaccines (with a residual pathogenic effect) [3,10]. Therefore, the best strategy to protect countries at risk of RVFV introduction is the implementation of regional monitoring and control programmes in endemic countries, as well as the establishment of early warning systems in the countries at risk. The Egyptian authorities implement both measures to prevent the introduction of RVFV (vaccination of imported animals, quarantines and testing of a proportion of imported animals) and measures to prevent the spread (vaccination of the Egyptian susceptible population). If those measures were effective, there would be no risk of RVFV introduction and spread, however, their efficacy has not been evaluated, and some deficiencies have been identified in the past [6]. Given the limitation of resources, risk-based methods (i.e. focusing on those areas and/or practices that pose the highest risk) may be considered as a cost-effective alternative for the surveillance of RVF in Egypt. Many of those risk-based methods are data-driven, which rely on comprehensive information about disease-associated events. In a data-scarce context, knowledge-driven methods, which rely on the previous information on the factors associated with disease occurrence, offer an alternative approach to identify areas at risk [11].

The **main objectives of this study** were:

1. To understand the structure and patterns of the legal trade of live animals into Egypt, and then how those animals are distributed throughout the country.
2. To evaluate the risk of RVFV transmission within Egypt (risk-mapping) by means of a geographical information system (GIS)-based multi-criteria evaluation (MCE) approach.
3. To combine animal trade data and risk of RVFV transmission data to identify those areas and periods in which the introduction of RVFV is more likely.

That will allow the improvement of the surveillance and control measures currently being implemented by the Egyptian Authorities, and make a better use of the resources available. Improvement of RVFV surveillance should allow the early detection of the disease, while the improvement of control measures should help prevent cases of RVFV infection in both animals and humans.

## Materials and methods

### Understanding the legal trade pattern of live cattle and camels into and within Egypt

#### Legal trade of live animals into Egypt

Animal trade data, including date (month and year), number of animals, and country of origin, for all the batches of cattle and camels legally imported into Egypt between 2012 and 2015 was provided by the Quarantine & Inspection Department (QID) of the Ministry of Agriculture and Land Reclamation of Egypt (MALRE). Only cattle and camels from two countries, Ethiopia and Sudan, were legally imported during that period. All cattle and camels imported into Egypt are quarantined, and data on the quarantine facilities in which the imported lots of animals was also provided by the QID.

#### Legal trade of animals within Egypt

All cattle imported into Egypt are slaughtered in the facilities available within the quarantine stations (i.e. they are not allowed to stay in the country as live animals). In contrast, camels, once released from the quarantines, may be sent to slaughterhouses or to animal markets. Data on the location of slaughterhouses and animal markets were provided by the Department of Epidemiology of the Ministry of Agriculture and Land Reclamation of Egypt (DE-MALRE). For animal markets, the DE-MALRE also provided information on the characteristics of the 273 animal markets present in Egypt, collected with the objective of ranking their risk in relation to the potential presence of exotic diseases, and allow for targeted surveillance and control. This ranking was initially developed for Foot and Mouth disease, but was adapted for RVF as follows:

#### Characterization of animal markets

The ranking of animal markets in relation to their risk of RVFV introduction was carried out according to a set of criteria. For each of those criteria, a risk score was assigned, so that, the higher the numerical score, the higher the risk. The criteria used for the evaluation were:

- Size: Average number of animals per operating day. The scores were: 1 for up to 1000 heads, 2 for between 1000 and 2000 heads, 3 for between 2000 and 5000 heads, 4 for between 5000 and 10000 heads, and 5 for more than 10000.
- Connectivity: If animals traded in the market originated within the same district, the score for connectivity was 1. If animals originated from the same governorate, the score was 2, and if animals originated from different governorates, the score was 3.
- Animal species traded: Camels was considered the key species in relation to the risk of RVFV introduction. In addition, the simultaneous presence of camels with animals of other species susceptible to RVFV (i.e. cattle, sheep and goats), which potentially had different origins, was also considered a risk factor. Therefore, a score of 4 was given to markets with camels plus other susceptible species, 3 to markets exclusively for camels, 2 to markets with 2 or more susceptible species (excluding camels) and 1 to markets with just one species susceptible to RVFV.
- Number of operating days. The scores were: 1 if the market opened a single day per week, 2 if the market opened 2–3 days per week, and 3 if the market opened 4 or more days per week.
- Potential presence of smuggled animals: Inquires carried out by the Egyptian Veterinary Services (EVS) revealed that some illegal trade of sheep and goats occurs through the border between Sudan and Egypt. Therefore, animal markets which trade sheep and goats, and are located in the governorates bordering Sudan were given a score of 2, while the others were given a score of 0.
- Key camel markets: To account for other factors not considered within the previous criteria, an extra 3 point score was assigned to the two main camel markets according to the opinion of the EVS.

Data on the location of all the camel slaughterhouses authorized in Egypt, as well as information on the number of animals slaughtered in each of them, was also provided by the DE-MALRE.

#### Measures to prevent the introduction of RVFV into Egypt and the transmission within Egypt

Information on the preventive measures and surveillance strategies implemented in Egypt were provided by the DE-MALRE. Preventive measures include the vaccination of both autochtonous and imported animals of the susceptible species against RVFV. The type of vaccine used is the RVFV inactivated vaccine (Zagazig H501 strain) produced by the Egyptian Veterinary Serum and Vaccine Research Institute (VSVRI) (http://www.vsvri-eg.com/Products/ProductsAnimal%201-4%20inactivated%20Rift%20Valley%20Fever%20Vaccine.html).

### Mapping the risk of RVF

The spatial risk of RVFV transmission within Egypt was estimated using a GIS-based multi-criteria evaluation (MCE) approach [12], which allows to combine the (spatial) data on the different factors that may influence the risk of a given disease. In the case of RVF it is essential to account for the distribution of both the susceptible hosts and the vectors of RVFV [13]. All layers were transformed into a raster format, with 1 km × 1 km spatial resolution and the common UTM 35N projection. R software [14] was used for both the analyses and generation of maps.

#### Mapping the distribution of susceptible hosts of RVFV

**Fig 1:**
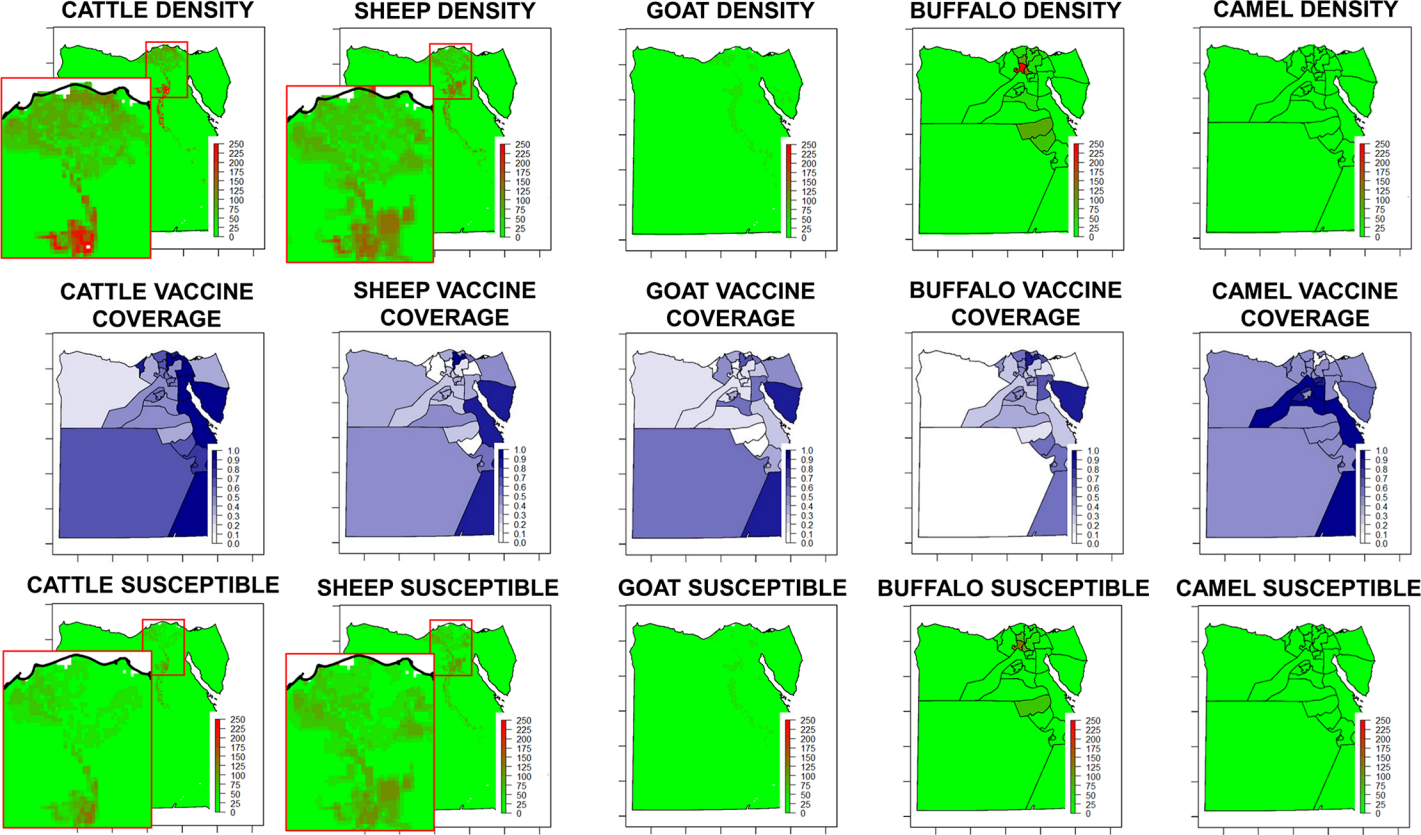
Distribution by species (density of animals, vaccine coverage and density of susceptibles). Upper row: density of cattle, sheep, goat and buffaloes (animals per square kilometer). Middle row: vaccine coverage for cattle, sheep, goat and buffaloes. Lower row: density of susceptible cattle, sheep, goat and buffaloes (animals per square kilometer).

Cattle, sheep, goats, buffaloes and camels were considered as the species susceptible for RVFV. Cattle, sheep and goats density maps were obtained from the FAO-Gridded Livestock of the World modelled data [15]. For buffaloes and camels, as density raster maps were not available, the data on the number of animals by governorate was obtained from OIE-WAHID, densities were calculated and maps were rasterized. Density maps for the different species (cattle, sheep, goats, buffaloes and camels) are shown in Fig 1-upper row. All animals of the susceptible species (cattle, sheep, goats, buffaloes and camels) are meant to be vaccinated twice a year (MALRE personal communication). Data on the number of vaccine doses administered the first and second semesters of 2014 and 2015 by animal species and governorates were provided by the ED-MALRE. For the year 2015, the vaccine coverage (i.e. the proportion of animals correctly vaccinated, that is, that received two vaccine doses) by species and governorates were calculated taking into account the censuses by species for the different governorates (Fig 1-middle row). By multiplying the animal density data layer by the vaccine coverage layer, we obtained a layer of the density of susceptible animals by species (Fig 1-lower row). Then, the weights for the relative susceptibility of the different host species (see below the determination of weights) were applied, and the results combined to produce a map to represent the density of all susceptible hosts. Finally, that map was standardized, i.e. put in a continuous scale from 0, equivalent to the minimum value (density), to 1, equivalent to the maximum value (density). Standardization allows the combination of layers with different characteristics/units to produce the final risk maps.

#### Mapping the distribution of *Cx*. *pipiens*

*Culex pipiens* has been traditionally considered the main vector of RVFV in Egypt [16], and recent entomological studies seem to confirm that [17]. In fact the competence of Egyptian *Cx*. *pipiens* for RVFV transmission was demonstrated experimentally [18]. Other species potentially involved in RVFV transmission is *Cx*. *antennatus*, considered by Gad and collaborators [19] as an important secondary vector of RVFV in Egypt. *Cx*. *antennatus* is found in every governorate of Egypt, and their primary breeding include flooded areas, rice fields and irrigation channels [20].

We mapped *Cx*. *pipiens* presence in Egypt based on the expected suitability of the land cover categories for the presence of this vector (values of 1 for presence and 0 for absence) estimated on the basis of a literature review and the opinion of experts [13]. Given that the same data was not available for *Cx*. *antennatus*, we used the distribution *Cx*. *pipiens* to represent the distribution of vectors competent for RVFV transmission. Even though other species may also be involved in RVFV transmission, in particular *Cx*. *antennatus*, which was the most abundant species in Kafr el Sheikh governorate in the 2003 epidemic, its primary breeding areas coincide with those of *Cx*. *pipiens*, and therefore would be included in the vector distribution map [16].

Given that estimates by Tran and collaborators [13] were based on Corine land-cover categories, which are not available for Egypt, the corresponding categories from FAO-GeoNetwork land cover were extrapolated from the South of Europe, and used to map the distribution areas of *Cx*. *pipiens* in Egypt. If a FAO-GeoNetwork land cover category corresponded to a single Corine land cover category, its value for the presence of *Cx*. *pipiens* was that of Corine land cover category (0 or 1). If a FAO-GeoNetwork land cover category corresponded to more than one Corine land cover category, its value for the presence of *Cx*. *pipiens* was the mean value for the corresponding Corine land cover categories.

Besides, in a recent study modelling the distribution of *Cx*. *pipiens* in arid and semi-arid regions of the Middle East and North Africa, Conley and collaborators [21] evidenced that human population density and seasonality of vegetation indexes were powerful predictors of *Cx*. *pipiens* occurrence in those areas. In particular, *Cx*. *pipiens* occurrence evidenced a strong negative association with the standard deviation (SD) of the enhanced vegetation index (EVI), that is, with the variation (seasonality) of the EVI. This suggests that *Cx*. *pipiens* habitat consists mainly of areas of high primary productivity, maintained with very little change throughout the year [21]. Monthly estimates of the SD of the EVI for the year 2015 were derived from Moderate Resolution Image Spectroradiometer (MODIS) imagery from the Terra Satellite, and were used to capture those seasonal variations in *Cx*. *pipiens* presence in Egypt. On the other hand, data on human population was obtained from the Gridded Population of the World version 4 [22].

The map of *Cx*. *pipiens* presence based on land cover, the map of human population and the maps of SD of the EVI (monthly values and also average for the whole year) were all standardized and combined to produce the map of the overall vector suitability (either monthly values or average for the whole year). Combination of layers was carried out with the criteria that when *Cx*. *pipiens* presence based on land cover was 0, the overall vector suitability was also 0 (regardless of the values of human population and SD of the EVI).

#### Evaluation of the seasonality in the distribution of *Cx*. *pipiens*

In order to evaluate the seasonal variability in the distribution of *Cx*. *pipiens*, monthly vector distribution maps were created using the monthly estimates of the SD of the EVI for the year 2015. Then, 10,000 random sampling points were generated, and the estimates of *Cx*. *pipiens* distribution for the different months, as well as the average value for the year, were obtained. As in Egypt for most of the country the probability of vector presence will be zero (desert areas), for most of the 10,000 random points the value would be zero, and that will compromise the probability of detecting a seasonal changes of the risk in the areas where risk is not zero. To avoid that, the areas of very low probability of vector presence (lower than 0.02) were excluded for the selection of the random points. Besides the 10,000 random points selected, the seasonal variability in the distribution of *Cx*. *pipiens* was also evaluated in the areas around (buffer of 5 kilometers) the quarantine premises, the main markets (the 51 riskiest markets) and the camel slaughterhouses, i.e. the specific locations where the risk of RVFV introduction and further transmission is expected to be highest.

For each location and each month, the variability in *Cx*. *pipiens* favorability (i.e. suitability) was calculated as:

$${Var}_{i,j}=abs\left(\frac{{Fav}_{i,j}-{Fav}_{j}}{{Fav}_{j}}\right)$$

where *Fav_i,j_* represents *Cx*. *pipiens* favorability in month *i* and location *j*, and *Fav_j_* represents *Cx*. *pipiens* favorability at location *j* for the whole year, and *Var_i,j_* is obtained as an absolute value.

#### Mapping the risk of RVFV transmission by vectors

Once obtained, the standardized maps of the distribution of susceptible hosts of RVFV and of competent vectors of RVFV were combined to produce a map of the risk of RVFV transmission by vectors.

#### Standardization and selection of membership functions

As in Stevens and collaborators [11], the risk was assumed to be proportional to the density of animals up to a certain level, above which risk was assumed to remain constant. That assumption avoids the underestimation of all the remaining values of density. Therefore, the relationship between density and risk was described with a monotonically increasing membership function shape, and a linear membership function type. The upper density limit was set at the percentile 99 of the density. The same type of relationship was established between human population density and suitability of vectors.

#### Determination of weights

The weights of the different factors that influence the risk of RVFV transmission by vectors were determined by means of the paired comparison method developed by Saaty [23]. The relative importance of each of the *n* factors relative to every other factor was evaluated using a 9-point reciprocal scale, in which 1 represents equal importance and 9 extreme importance, while 1/9 would represent of extremely low importance [23]. That results in a *n* x *n* matrix of pairwise comparisons, and the dominant eigenvector of that matrix corresponds to the vector of relative weights for the *n* factors.

#### Sensitivity analysis

In order to evaluate the effect of a change in the weights applied to each of the risk factors on the risk of RVFV transmission, a sensitivity analysis was carried out [24]. For each of the factors, its weight was increased and then decreased by a certain proportion (+/-10%, +/- 20% and +/-30%), while the weights for the other factors were proportionally increased or decreased (so that the sum of weights added to 1), and the values of the risk of RVFV transmission as a consequence of those changes were measured at 10,000 random locations. Then, a linear regression model was fitted to the data, and the contribution of each factor to the variation of the risk of RVFV transmission was calculated as the ratio of the sum of squares related to that factor on the total sum of squares of the model.

#### Validation of risk maps

In order to validate the risk of RVFV transmission map, data on any previous outbreak of RVFV occurred in Egypt for which the location was available was collected from several sources. For the 1977 epidemic data on the location of several outbreaks which affected humans and animals were reported by Meegan [5]. Affected locations included the villages of Bilbeis, Zagazig and Inshas (in Sharqia governorate), El Khanka (in Qalyubia governorate) and Imbaba (in Giza governorate). For the 1993 epidemic several outbreaks affecting cattle, sheep and goats were reported to the World Organisation for Animal Health [25] in the villages of Kom Ombo, Edfu and Al Balyana (in Aswan governorate). In the 1997 epidemic, outbreaks in cattle and sheep were reported in the villages of Daraw (in Aswan governorate) and Manqabad and Durunkah (in Asyut governorate) [26]. In 2003, repeated cases of RVF in humans were reported to the World Health Organization [27] in Seedy Salim district (in Kafr Al-Sheikh governorate). Besides, from 297 pools of female mosquitoes collected in Kafr Al-Sheikh governorate, RVFV was isolated in 3 pools of *Culex antennatus* (Becker) from 2 different sites. In 2012, an outbreak of RVF in cattle was reported by FAO in the village of Al Khwajat (in Faiyum governorate), although the case was not confirmed by the Egyptian Authorities [28]. In total, 16 outbreak locations were used for the validation of the risk map.

### Combination of animal trade data and risk of RVFV transmission data

Besides the favorability for transmission in a given area, the actual risk of RVF occurring in that area is determined by the probability of the virus being introduced into that location. Given that in Egypt imported animals are quarantined on specific premises on arrival to the country, and that transmission within those quarantine stations and to animals in their surroundings may occur, those will be the risk locations of primary interest. No measures to protect animals from mosquitos are taken on those quarantines.

After quarantine, imported camels may be taken to markets or to slaughterhouses, where transmission within the premises and to animals in their surroundings may also occur, and therefore those will be the risk locations of secondary interest.

Studies on the dispersal of *Cx*. *pipiens* in urban habitats found maximum flight ranges of 1.98 km [29] and 2.48 km [30]. However, given the low probability of recapturing a marked mosquito at large distances from the point of release, we used a conservative radius of up to 5km for the buffer area. We also assessed the effect of changing the size of the radius on the risk of RVF transmission.

## Results

### Understanding the legal trade of cattle and camels into and within Egypt

#### Legal trade of cattle and camels into Egypt

**Fig 2:**
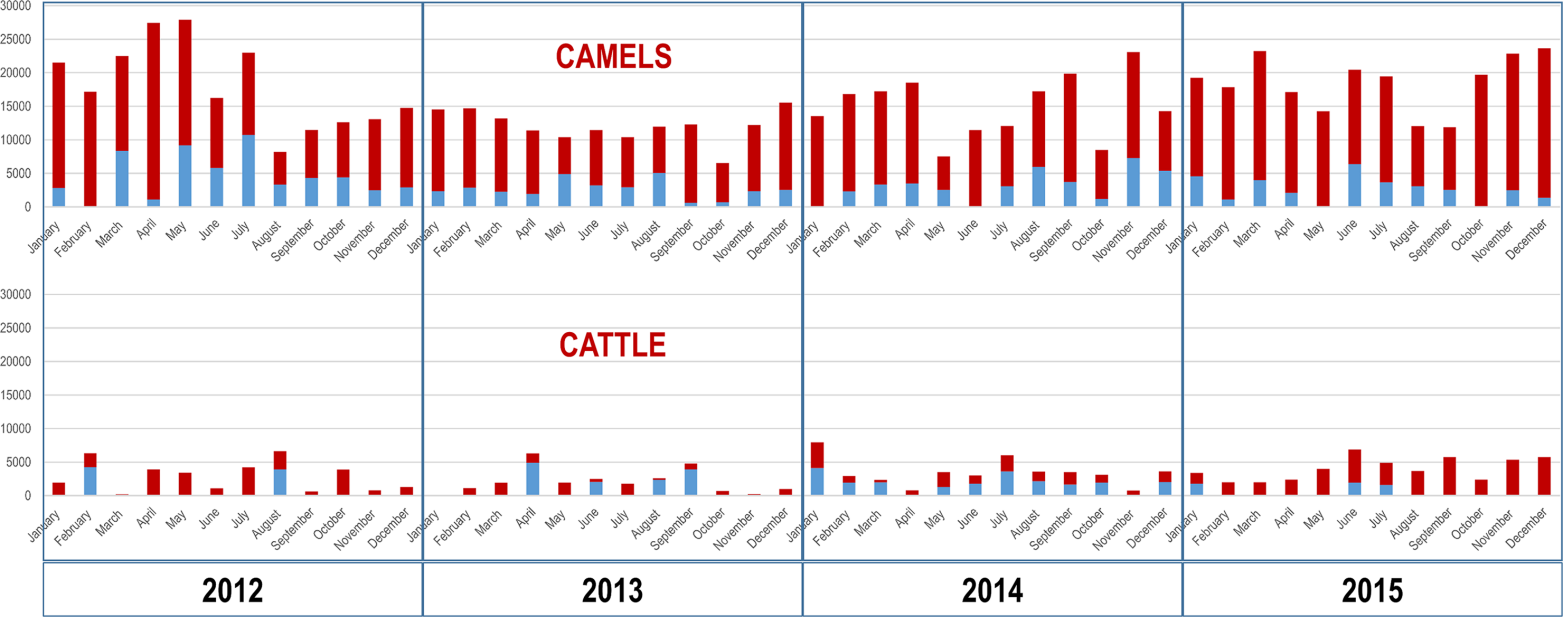
Importation of camels and cattle. Monthly importation into Egypt of camels and cattle between 2012 and 2015. In red animals imported from Sudan, and in blue animals imported from Ethiopia.

**Camels**. During the period 2012–2015, 762,291 camels were legally imported into Egypt, 79.4% of which came from Sudan, and the rest (20.6%) from Ethiopia. The temporal pattern (Fig 2) evidences a continuous flow all the months between 2012 and 2015. However, throughout this period there were some significant variations. From January 2012 to July 2012, the average number of camels imported per month was above 20,000. After that, and until the end of 2013, the number decreased to about 12,000. Then, importations progressively increased to about 15,000 in 2014 and 18,000 in 2015 (Fig 2). That increase coincided with the intensification of the trade with Sudan, as the number of camels imported from Ethiopia actually decreased. No seasonal pattern was observed in the amount of camels imported into Egypt throughout this period.

**Fig 3:**
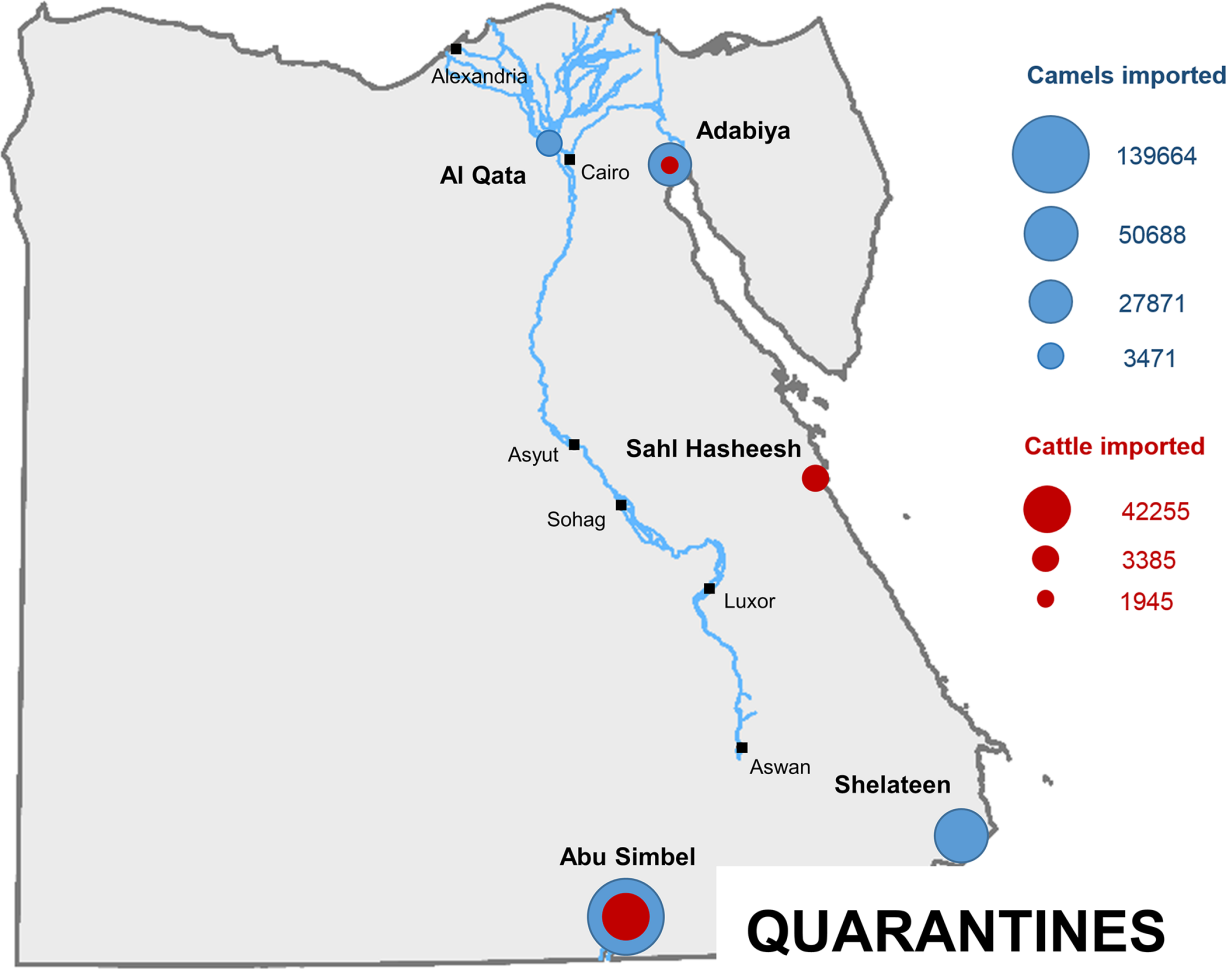
Legal trade routes for cattle and camels imported into Egypt from Sudan and Ethiopia.

a) Importation of camels from Sudan: of all the camels imported in 2015, 139,664 (63% of the total) arrived from Western Sudan to Abu Simbel quarantine in South-Central Egypt (Fig 3). Another 50,688 (23% of the total) arrived from Eastern Sudan to Shalateen quarantine in South-Eastern Egypt (Fig 3).

b) Importation of camels from Ethiopia: all camels imported from Ethiopia in 2015 (31,342, 14% of the total) arrived via Djibouti to Adabiya port in North-Eastern Egypt (Fig 3). Of them, 27,871 remained in Adabiya quarantine (Fig 3), and 3471 were transferred to Al Qata quarantine in Giza governorate (Fig 3).

**Cattle**. Of the 148,943 cattle imported into Egypt between 2012 and 2015, 67% came from Sudan and 33% from Ethiopia (Fig 2). For 2012 and 2013 the temporal pattern was quite irregular with some months with very low or no importations and others with more than 6000 cattle imported (Fig 2). In 2014 and 2015 the pattern became more regular, and there was also a progressive increase in the number of animals imported, from about 2000 cattle per month in 2013, to about 3500 in 2014 and 4000 in 2015. That increase coincided with the intensification of the trade with Sudan and the decrease of the trade with Ethiopia (Fig 2). In contrast to camels, there seems to be a seasonal pattern in the importation of cattle into Egypt, mainly in the period between July and September, when importations were about 29% higher than the average for the year.

a) Importation of cattle from Sudan: all the cattle imported in 2015 from Sudan (42,255), 89% of the total cattle imported into Egypt, arrived from Sudan to Abu Simbel quarantine (Fig 3).

b) Importation of cattle from Ethiopia: all the cattle imported from Ethiopia in 2015 (5530) arrived via Djibouti. Of them, 1945 went to Adabiya port and were transferred to Adabiya quarantine (Fig 3). The remaining 3385 arrived to Safaga Port and were transferred to Sahl Hasheesh quarantine, in Central-Eastern Egypt (Fig 3).

#### Legal trade of cattle and camels within Egypt

Once released from quarantine, imported camels may be sent to a slaughterhouse or be transported to animal markets. In contrast, all imported cattle have to be killed in the slaughterhouses within the quarantine premises.

#### Characterization of animal markets

**Fig 4:**
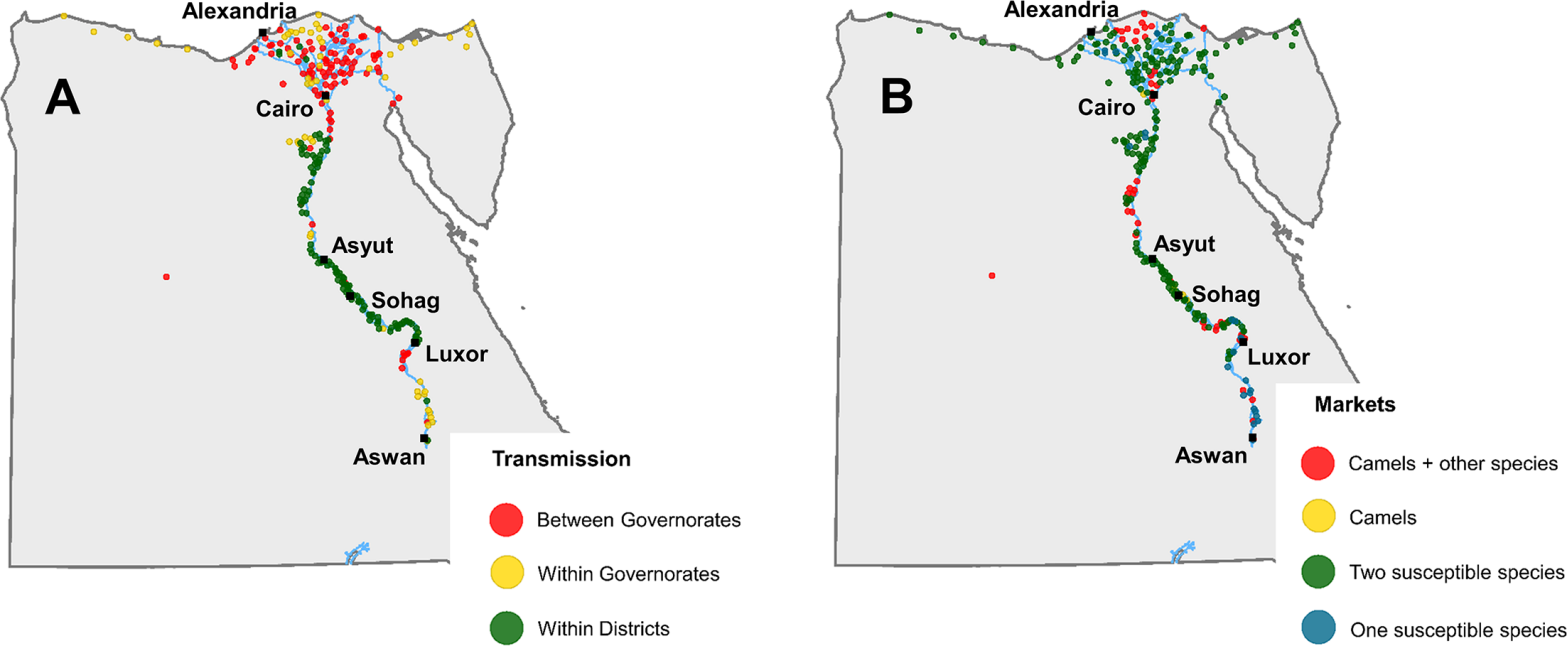
Location of the 273 animal markets by connectivity (A) and by RVFV susceptible species traded (B).

The 273 animal markets evaluated are distributed throughout Egypt, mainly in the areas beside the Nile river and its delta, although there are also animal markets alongside the Mediterranean coast, or even in the Sahara desert (Farafra market) (Fig 4). The characteristics of the animal markets according to the different criteria are summarized in Table 1.

**Table 1:** Characteristics of the animal markets description of risk scores for the different criteria and relative frequency.

	Score 1	Score 2	Score 3	Score 4	Score 5
**Size**	< 1000 heads	1000–2000 heads	2000–5000 heads	5000–10000 heads	> 10000 heads
	71%	15%	9%	4%	1%
**Connectivity**	Same district	Same governorate	Different governorates		
	41%	21%	38%		
**Species traded**	One species	Two or more species	Camels	Camels & other species	
	10%	71%	2%	17%	
**Operating days per week**	1 day	2–3 days	> 3 days		
	97%	3%	1%		
**Potential presence of smuggled animals**		Yes			
		6%			

The majority of markets that trade animals between different governorates are located in the triangular area between Alexandria, Cairo and Port Said (Fig 4A), while the majority of markets to the south of Cairo are local markets (that only trade animals from the same district). The markets that trade camels (key species) are located all along the Nile river basin (Fig 4B). Only 17 of the 273 markets (6%) were located in the governorates bordering Sudan (16 in Aswan and 1 in the New Valley).

**Fig 5:**
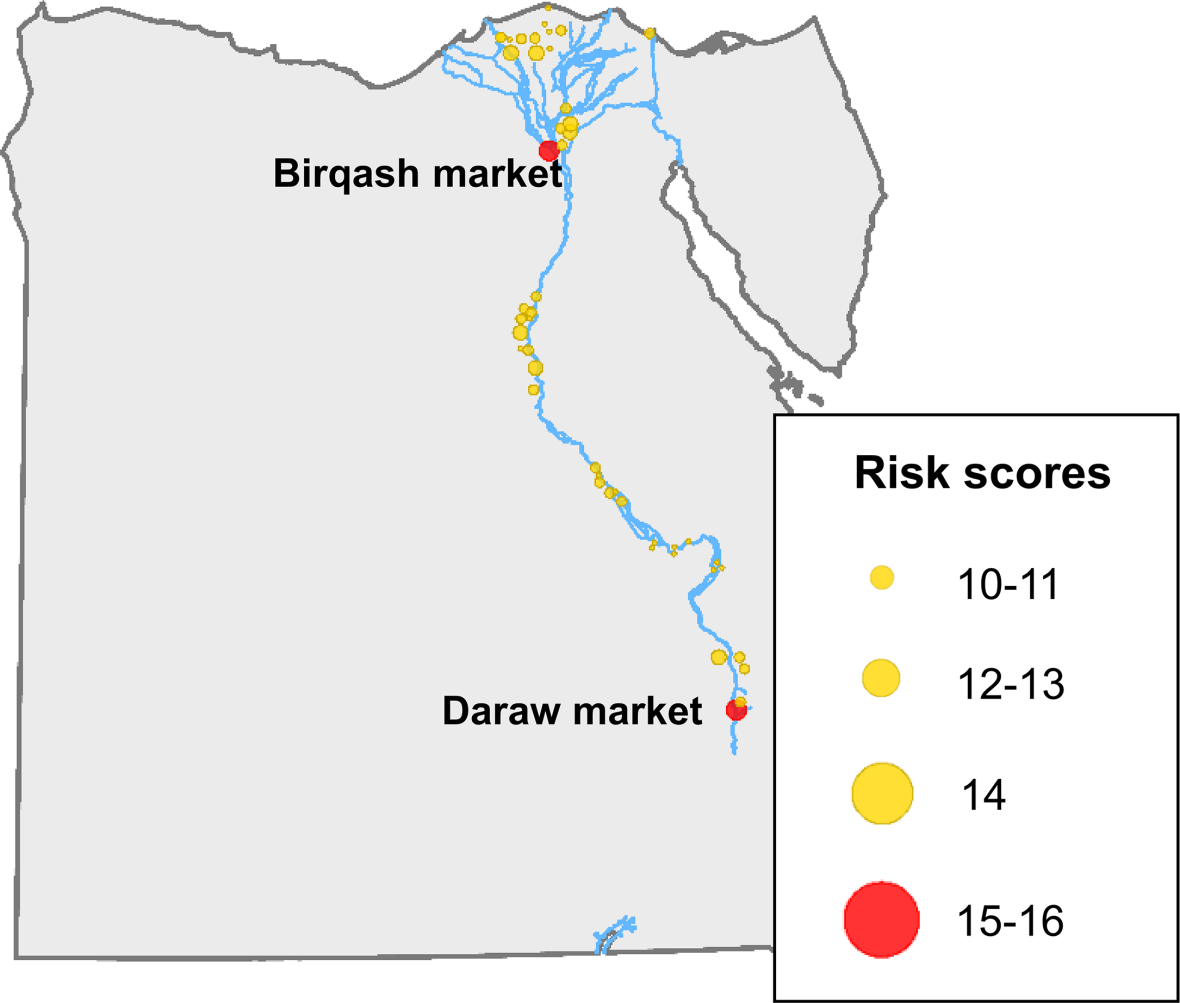
Location of the 51 animal markets with the highest risk for RVFV transmission (risk score above 10).

The RVFV risk-ranking of animal markets varied between 5 and 16 (out of a maximum of 20), with a mean value of 7.9. The markets with the highest scores were mainly located in the area between Cairo and Alexandria, to the north of Asyut and in the proximity of Aswan (Fig 5). The two markets with the highest score were Birqash market (northwest of Cairo) and Daraw market (north of Aswan).

#### Characterization of camel slaughterhouses

**Fig 6:**
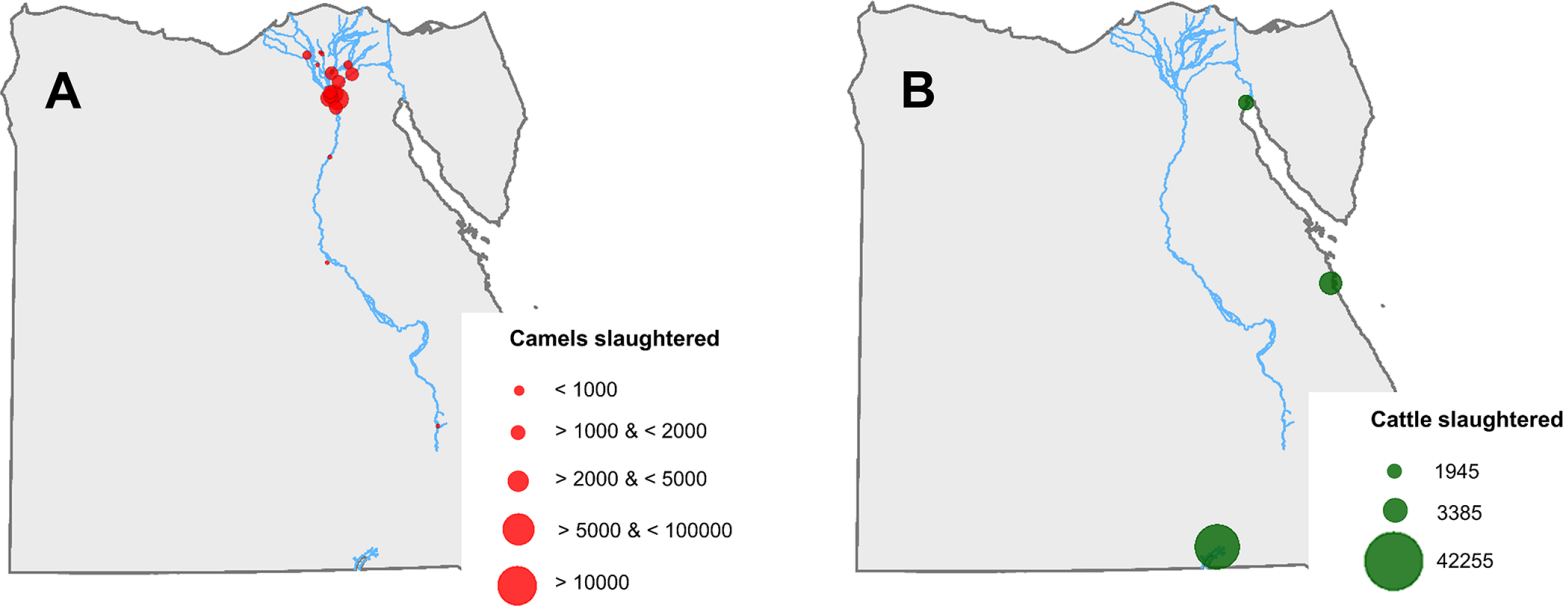
Location of the slaughterhouses by number of animals slaughtered for camels (A) and for cattle (B).

There are 18 slaughterhouses authorized for camels in Egypt, mainly located in the proximity of Cairo (Fig 6A). The number of camels slaughtered, according to the 2014 data provided by the DE varied between 12,823 and 135,000.

#### Characterization of cattle slaughterhouses

All cattle imported into Egypt are slaughtered within the quarantine premise to which they arrive. Therefore, the 42,255 cows imported from Sudan in 2015 were slaughtered in Abu Simbel quarantine (Fig 6B). Of the 5530 cows imported from Ethiopia in 2015, 1945 were slaughtered in Adabiya quarantine, and the remaining 3385 in Sahl Hasheesh quarantine (Fig 6B).

#### Measures to prevent the introduction of rvfv into Egypt

**Camels.** Camels from Sudan are quarantined in Abu Simbel or Shelateen facilities. On arrival they receive one dose of vaccine against RVFV. They are maintained in quarantine for 3 days, after which they are transported to animal markets or to slaughterhouses. Camels imported from Ethiopia arrive to Adabiya port from where they are transferred to Al Qata or to Adabiya quarantines. They arrive via Djibouti, where they receive a first dose of vaccine 7 days prior to shipment. A second dose of the vaccine is administered once they are in the quarantine facility in Egypt, where they are maintained for 10–16 days. In addition, about 10% of the camels were tested against RVF by RT-PCR. All the camels imported from Sudan and Ethiopia tested in the last 5 years were negative (QID personal communication).

**Cattle**. Cattle imported from Sudan receive the first dose of RVFV vaccine in Sudan under the supervision of the EVS. Cattle imported from Ethiopia arrive via Djibouti, where they receive the first dose of vaccine. And once in the corresponding quarantine in Egypt, about 75% of cattle receive a second dose of the vaccine to prevent transmission in case quarantine is prolongued (QID-MALRE, personal communication). Cattle remain in the quarantines just for a few days, until they are slaughtered.

#### Measures to prevent the transmission of rvfv within Egypt

The vaccination of the susceptible population is carried out on yearly basis by the EVS.

#### Vaccination of the Egyptian animal population against RVFV

In Egypt, all the animals of the susceptible species (cattle, sheep, goats, buffaloes and camels) are meant to be vaccinated twice a year with RVF inactivated vaccine (Zagazig H501 strain).

The estimation of the vaccine coverage of the Egyptian camel population is complicated because the data provided by the ED-MALRE includes also the vaccine doses used on imported camels. However, that would not have a significant effect on the results as the resident camel population in Egypt is really small as compared to the imports. In 2015, the year we used to account for the vaccine coverage in our model, 7.3 million doses of vaccine were administered as compared to 5.9 million in 2014. The analysis of the vaccine coverage data evidences a great degree of heterogeneity in the vaccination among species and areas (Fig 1). Taking into account that 2 doses of vaccine are needed, cattle had the highest level of vaccine coverage with about 60%, while the coverage was about 35% for buffaloes and about 30% for sheep and goats. There were also significant differences among areas, including some governorates with a high census of domestic ruminants and very low vaccine coverages. For example vaccine coverages in cattle were 20% in Asyut, 30% in Sharqia, 30% in Beheira, or 30% in Beni Suef, while in other species coverage was even lower (Fig 1).

### Mapping the risk of RVF

#### Mapping the distribution of susceptible hosts of RVFV

As the density of animals and the level of vaccine coverage vary among species and areas, their combination results in a heterogeneous density of susceptible animals (Fig 1). Cattle has the highest density, but also the highest vaccine coverage, so other species, for example sheep and goats in Sohag, or goats and buffaloes in Asyut, contribute more to the risk of RVFV transmission.

**Fig 7:**
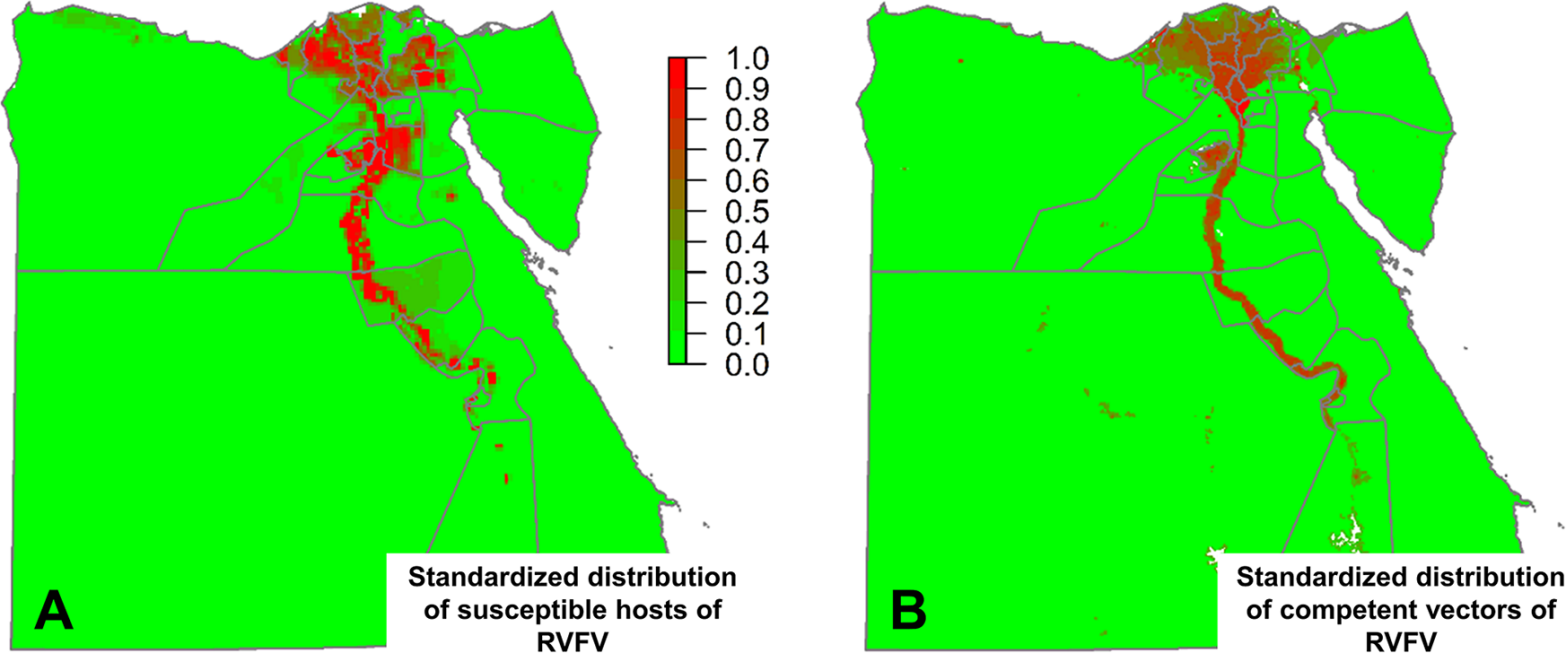
Standardized distribution of susceptible hosts of RVFV (A), and standardized distribution of of competent vectors of RVFV (B).

Sheep and goats are traditionally considered more susceptible for RVFV [31], and therefore those species were assigned a value of 3 (i.e. moderate importance) in relation to the other species. The weights for the relative susceptibility of the different host species, determined by the paired comparison method developed by Saaty [23] were 0.32 for sheep and goats and 0.12 for cattle, buffaloes and camels. The standardized distribution of susceptible hosts of RVFV (all species) shows a pattern which is mainly coincident with the areas surrounding the Nile river, although there are some significant variations in density (Fig 7A).

#### Mapping the distribution of competent vectors of RVFV

Most of the surface of Egypt corresponds to bare soil land cover class, where the natural vegetation is absent or almost absent. Bare soil land cover is not suitable for the presence of *Cx*. *pipiens* [13] and therefore the distribution of the vector is restricted to only a small proportion of the surface of Egypt (wet areas surrounding the Nile river) (Fig 7B). Within suitable areas, *Cx*. *pipiens* density is proportional to the human population density and the seasonality of vegetation indexes. Given this proportionality to the density of the human population, the vector map produced will be a proxy of the potential risk of RVFV transmission from mosquitos to humans (zoonotic risk).

In order to evaluate whether there was a seasonal component in the distribution of *Culex pipiens*, and therefore in the risk of RVFV transmission, the (standardized) values of *Cx*. *pipiens* favorability were extracted at 10,000 random locations from areas where values were not zero, as well as in buffer areas around the quarantines, the main animal markets and the camel slaughterhouses. The results (Table 2) indicate that in general there was not much variation in *Cx*. *pipiens* favorability throughout the year (mean variability for random points of 0.014), although there were some specific locations where *Cx*. *pipiens* favorability more than doubled. In risky locations (quarantines, markets and slaughterhouses) there was not much variability in *Cx*. *pipiens* favorability throughout the year: mean variability of 0.027 for markets, 0.026 for camel slaughterhouses, and 0.012 for quarantines. The maximum variability was found in markets (0.15) and camel slaughterhouses (0.12), while the maximum variability in quarantines was only 0.05.

**Table 2:** Mean and maximum variability in 10,000 random points (in the areas where risk is not zero), and at the main markets (51), camel slaughterhouses and quarantines.

	*mean*(*Var_i,j_*)	*max*(*Var_i,j_*)
Random Points	0.014	1.12
Main Markets	0.027	0.15
Camel Slaughterhouses	0.026	0.12
Quarantines	0.012	0.05

#### Mapping the risk of RVFV transmission by vectors

**Fig 8:**
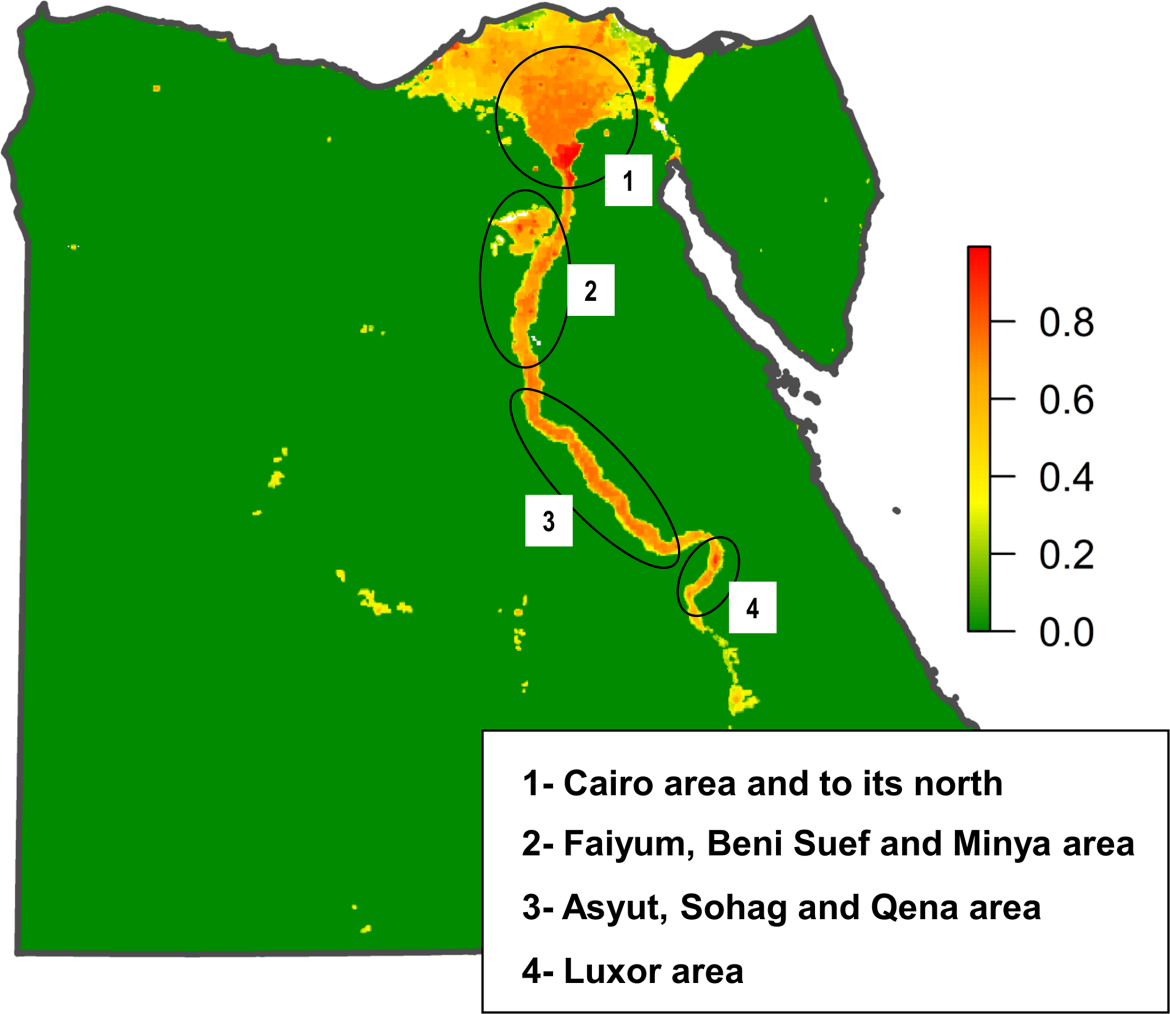
Standardized map of the risk of RVFV transmission by vectors in Egypt. Circles indicate the areas at the highest risk: Cairo area and to its north (1), Faiyum, Beni Suef and Minya area (2), Asyut, Sohag and Qena area (3), and Luxor area (4).

In Egypt the distribution of susceptible hosts and competent vectors of RVFV overlap in most areas and that results in that the areas at risk of RVFV transmission is restricted to the wet areas surrounding the Nile River and its delta. However, within the areas where RVFV transmission by vectors was estimated as possible, there are significant differences in the risk (Fig 8). The areas identified with the highest risk are the Cairo area and to its north (Fig 8-1), Faiyum and Beni Suef area (Fig 8-2), Asyut, Sohag and Qena area (Fig 8-3), and Luxor area (Fig 8-4).

#### Sensitivity analysis

The linear regression model indicates that the risk of RVFV transmission by vectors was mainly explained by the distribution of *Cx*. *pipiens* (*β* coefficient = 0.42; *p-value*<0.001) and to a lesser extent by the density of susceptible goats (*β* coefficient = 0.14; *p-value*<0.001). The remaining host species (cattle, sheep, buffaloes and camels) were not significantly associated with the risk of RVFV transmission by vectors.

#### Validation of risk maps

**Fig 9:**
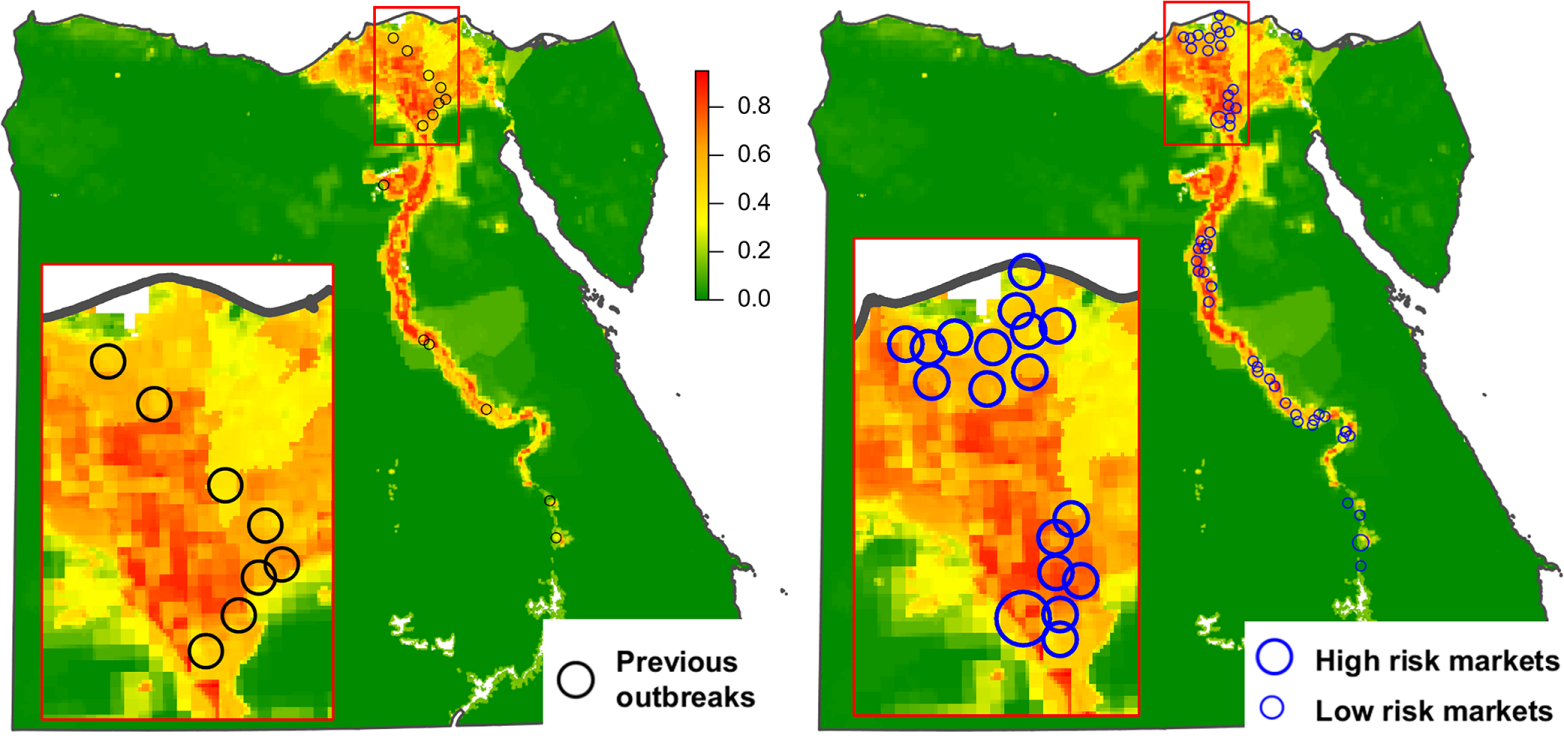
Risk of RVFV transmission by vectors at the locations of previous outbreaks and at the main animal markets. (A) Risk of RVFV transmission by vectors at the locations where outbreaks from previous epidemics occurred. (B) Risk of RVFV transmission by vectors at the locations where the 51 main animal markets are located. Large blue circles represent the two markets with the highest risk (Birqash market in Giza governorate and Daraw market in Aswan).

The location of RVFV-positive cases in humans and animals, as well as RVFV-positive vectors from previous RVFV epidemics (for which the location was available) were used to validate the risk of RVFV transmission by vectors map (Fig 9A). The risk obtained at the location of outbreaks (considering a radius of 1 kilometer around each point) varied between 0.18 and 0.85, with a mean value of 0.49. Variation of the size of the radius (up to 5 kilometers) resulted in general in little variation of the risk, although there were some exceptions in which the risk could vary more than a 20%.

### Combination of animal trade data and risk of RVFV transmission data

**Fig 10:**
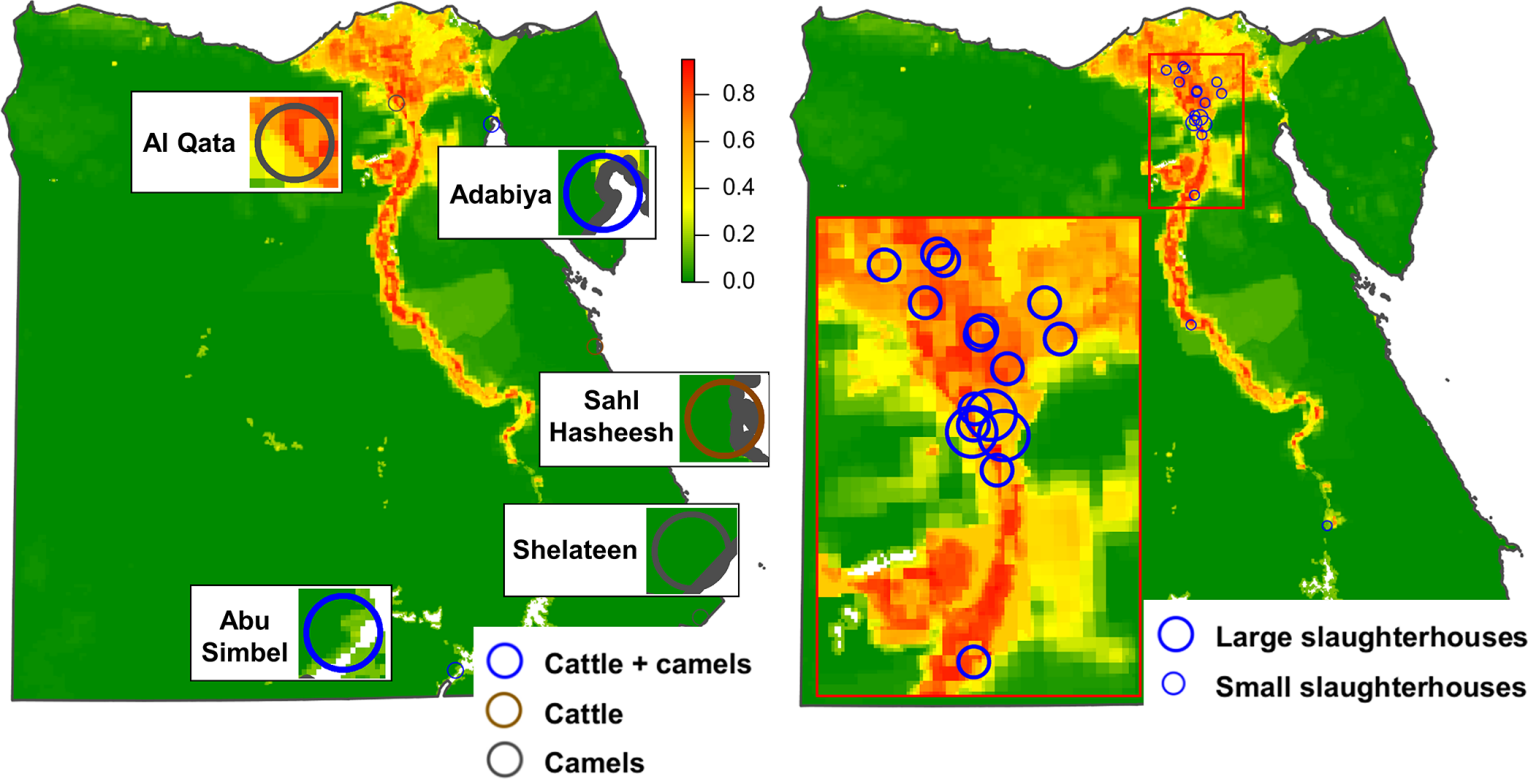
Risk of RVFV transmission by vectors at the locations of quarantines and camel slaughterhouses. Risk of RVFV transmission by vectors at the locations where quarantines are located (A). Risk of RVFV transmission by vectors at the locations where the camel slaughterhouses are located (B). Large blue circles represent the three slaughterhouses that kill the most camels.

Animals imported into Egypt are not randomly distributed throughout the country, instead they are transported to specific locations where the risk of RVFV transmission by vectors will be quite variable. First, they are taken directly to specific quarantine premises depending on the species and origins (Fig 10A). After quarantine, imported camels also follow an established movement pattern: they may be either be taken to slaughterhouses (Fig 10B) or to animal markets (Fig 9B). In contrast, all cattle are slaughtered within the quarantine facilities.

The summary estimates of the risks of RVFV transmission by vectors at those main locations after the importation of animals, i.e. markets, quarantines and camel slaughterhouses, considering buffers of either 5000 or 2000 meters, are shown in Table 3.

**Table 3:** Risk of RVFV transmission by vectors at the main locations after the importation of animals: Markets, quarantines and camel slaughterhouses considering either a buffer of 5000 meters or 2000 meters.

	5000 meters			2000 meters		
	Minimum	Mean	Maximum	Minimum	Mean	Maximum
High-risk markets (51)	0	0.57	0.81	0	0.58	0.82
All markets (273)	0	0.60	0.84	0	0.62	0.87
Quarantines (5)	0	0.17	0.70	0	0.20	0.81
Camel slaughterhouses (18)	0.15	0.59	0.76	0.15	0.60	0.81

Of the quarantines (Fig 3 and Fig 10A), Abu Simbel, where a significant proportion of the camels and all the cattle imported from Sudan arrived, seemed to have a low risk of RVFV transmission by vectors (between 0.04 and 0.08 depending on the buffer considered). For Shelateen quarantine, where camels imported from eastern Sudan arrived, the risk was 0. For Sahl Hasheesh, where cattle from Ethiopia arrived, the risk was extremely low (between 0 and 0.01 depending on the buffer). For Adabiya, where camels from Ethiopia arrived, the risk was also relatively low (between 0.07 and 0.14). In contrast, for Al Qata, where also camels from Ethiopia arrived, the risk of RVFV transmission by vectors was quite high (between 0.70 and 0.83).

Of the two markets with the highest risk-ranking score (Fig 5), Aswan had a risk of RVFV transmission by vectors relatively low (between 0.15 and 0.16), while Birqash market had risk of RVFV transmission by vectors quite high (between 0.70 and 0.74) (Fig 9B). Of the remaining high-risk markets there are 10 with risk of RVFV transmission by vectors estimates above 0.7. Of them, 7 are located in risk-area 1 in Fig 8, in the governorates of Gharbia (3), Qalyubia (3) and Monofia (1). The other 3 are located in Minya governorate (risk-area 2). In risk areas 3 and 4, there is only one high-risk market in each with risk of RVFV transmission by vectors above 0.5, in Qena and Aswan governorates, respectively.

The camel slaughterhouses (Fig 6A and Fig 10A) are mostly located in the proximities of Cairo, where conditions are favourable for RVFV transmission and therefore their risk are relatively high (Table 3). Of the three slaughterhouses that killed the most animals, Elbasateen had a risk 0.49 regardless of the buffer distance, while Elwarak had a risk between 0.47 and 0.50, and Kerdassa between 0.57 and 0.69.

## Discussion

Camel meat is an important source of cheap protein for the Egyptian people. However, as the resident camel population in Egypt is really small, 66,233 animals in 2015 (according to OIE data) and located mainly in remote areas, large number of camels need to be regularly imported into Egypt, the majority of which come from Sudan (86% of the total in 2015) and the rest from Ethiopia. The flow of camels is continouos throughout the whole year and the number of animals imported per year seem to have an increasing trend. In 2015, an average of 18,000 camels were imported per month.

During the first RVF epidemic in Egypt in 1977, mortality and abortions were reported in camels in Aswan governorate (in the south of the country), and RVFV was isolated from this species [32,33]. Therefore, even though the source of the outbreak could not be determined, the possibility of introduction of the disease from Sudan by camels was considered the most likely explanation. In the following epidemics (1993, 1994, 1997 and 2003), importation of camels, mainly from Sudan, was also considered as a possible source of introduction, although no proper epidemiological investigations were carried out, and alternative mechanisms were considered [6,26]. As a result, the role of camels in the epidemiology of RVFV remains unclear. In fact, it has been suggested that camels may be essential for RVFV transmission in some ecosystems where their density is high, which would allow viral amplification and transmission to alternative hosts, but not in others [34]. Furthermore, data on factors such as duration of viraemia, infectiousness to mosquitos, or the risk of infection of humans from contaminated products remain unknown, and further studies on camels would be required. Despite those uncertainties, camels are considered susceptible to RVFV, and the virus has been isolated from blood from healthy, naturally infected camels in Egypt and Sudan [32,35]. While RVFV infections in camels are usually mild or asymptomatic, abortion waves were observed in RVF outbreaks in Kenya and Egypt [34]. In the 2010 epidemic in Mauritania, clinical signs other than abortions were observed in camels, either a peracute form with sudden death within 24 hours; or an acute form with fever and systemic lesions, that resulted in death when haemorrhagic signs occurred [36]. RVFV infections in camels have been recorded in most sub-Saharan African countries, with prevalences ranging from 3.0 to 51.9 percent [34]. In fact, RVF is thought to be endemic in sub-Saharan African countries (including Sudan), with large epidemics occurring after heavy rainfall and flooding [9]. Phylogenetic analyses carried out in Sudan with RVFV strains from the 2007 and 2010 epidemics, indicated that they were the result of multiple introductions from eastern Africa [37]. Since then, there were evidences of circulation of RVFV during the inter-epidemic period: in 2014–2015, 9.6% of camels in Khartoum State (Sudan) had RVFV antibodies, including at least one positive born after the last reported epidemic [38].

Besides, camels may be involved in the introduction of other diseases such as MERS-CoV, which may cause severe disease in humans. MERS-CoV was detected in Egypt in camels imported from Sudan and Ethiopia [8], and seems to still be present in Ethiopian camels [39].

Camels provide food (milk and meat), fibre (wool and leather) and draft power (for transportation and cultivation) to human communities located in dry environments [34]. As a result, in some societies they are essential from a cultural and socio-economic point of view. The world camel population has steadily increased in recent years to reach almost 28 million heads in 2014 (according to FAO). Besides, camel farming practices are changing, with a significant increase of intensive production in peri-urban areas, which results in a closer contact with the human population [34]. As camels are used for trade allowing the connection distant populations, in particular in arid and semi-arid ecosystems, they may play a role in the large-scale dissemination of pathogens.

The number of cattle legally imported into Egypt, 4074 on average per month in 2015, is small as compared to camels. The flow of cattle became more regular by the second half of the 4-years period, coinciding with the intensification of the trade with Sudan. In contrast to camels, the data revealed a more seasonal pattern, with an increase of importations in the months of July, August and September, just before the major Islamic feast of Eid al-Adha (“Sacrifice Feast") in those years. Importation of cattle also poses a threat for Egypt, in fact, the epidemics of foot and mouth disease (serotype A) and of lumpy skin disease that affected Egypt in 2006 were both linked to the importation of animals from Ethiopia [40]. Increase of importations of cattle and camels into Egypt may be explained by the growing demand of meat. While between 2006 and 2015 the human population in Egypt grew a 19.9% [41], the population of ruminants and camels in the country only grew a 3.2% [42]. According to the World Bank projections, the human population in Egypt is expected to continue growing at a rate of almost 2% per year in the following decade, and therefore legal (and potentially illegal) trade of animals into Egypt is expected to increase.

After quarantine, camels may be transported to markets or slaughterhouses throughout Egypt. Those movements within Egypt would contribute the spread of RVFV in the event of introduction of the virus, as evidenced in other countries [43,44]. The EVS characterized the network of animal markets by collecting information on the location, but also on several characteristics of 273 animal markets distributed throughout Egypt. That combined with the slaughterhouse data allows having a basic picture of the animal movement patterns within the country, which offers an alternative approach in countries without the resources to have a comprehensive recording of animal movements. That information is very useful for planning risk-based surveillance strategies (i.e. target those markets where detection of a particular disease is more likely), with the advantage the criteria may be adapted to each specific disease. The collection of more comprehensive information on for example the number of animals by species or the origins and destinations of the animals that went across those markets, would allow a much clearer picture of the animal movement network, and is therefore recommended The animal market network in Egypt is mainly composed of small markets, which basically operate once a week. Interestingly, almost 40% of the markets traded animals from different governorates, which means that infectious diseases are likely to spread efficiently once introduced into the country. In relation to the species, 2% of markets traded exclusively camels, and a 17% further traded camels plus other species susceptible to RVFV. Most of the markets are located in the areas beside the Nile river and its delta, but there are also animal markets alongside the Mediterranean coast, or even in the Sahara desert (Farafra market). Of the 273 markets, only 17 were in the governorates bordering Sudan, where smuggling of small ruminants was considered more likely. The risk-ranking score for markets varied between 5 and 15, with a mean value of 7.9. The markets with the highest risk of RVFV transmission were in the proximities or within the main cities (e.g. Cairo, Minya or Aswan), while most of the camel slaughterhouses were near Cairo. That proximity to urban areas results in an increased risk of RVFV transmission to humans (zoonotic risk). In contrast to camels, all cattle imported into Egypt are slaughtered within the quarantines to which they arrive, which are located in remote areas, far from populated areas.

In order to prevent the introduction of RVFV by camels, the EVS implement a series of measures that include vaccination of imported animals, quarantines and testing of a proportion of them. However, the vaccination strategy is unlikely be effective, as camels imported from Sudan receive a single dose and camels imported from Ethiopia receive two doses, but with only 7 days difference. The inactivated vaccines against RVFV require a booster 3–6 months after initial vaccination, followed by yearly boosters [45]. Besides, the efficacy of the inactivated vaccine has not been tested in camels [45], and further research on that field would be needed. While it has been argued that the long distance that the camels from Sudan need to walk to reach the Egyptian border would be enough to prevent the importation of infected animals, there are still many uncertainties in relation to RVFV infection in camels, and in any case, that measure does not comply with OIE rules for importation of live animals [6]. On the other hand, means of transportation seem to be changing as at least some of the camels are currently transported on trucks rather than on foot (MALRE personal communication) and transmission (either direct or indirect) during the transport of camels cannot be ruled out. Seroconversions in Madagascar highlands evidenced the local circulation of RVFV in periods in which mosquitoes were rare or inactive, suggesting that other forms of transmission seem likely [46]. Even though camels are quarantined, for 3 days if coming from Sudan and 10–16 days if coming from Ethiopia, the efficiency of clinical detection of RVFV infection is limited by the fact that infections in camels are often unapparent. Besides, about 10% of the camels imported from Sudan and Ethiopia in the last 5 years were tested against RVF by RT-PCR, and were negative (MALRE personal communication). However, the sensitivity of such non-statistically based testing protocol for detecting the infection, if present, is questionable. While the measures currently implemented in camels are likely to reduce some of the risk of RVFV introduction into Egypt, their effect is likely to be limited. Similar conclusions may be drawn from the strategies implemented in cattle with the particularities that all cattle are slaughtered within the quarantine premises, where the risk of RVFV transmission by vectors was estimated to be very low. Therefore, vaccination of imported cattle is unlikely to have a significant effect on the reduction of the risk of RVFV introduction into Egypt.

On the other hand, to prevent the transmission of RVFV within Egypt, all the animals of the susceptible species (cattle, sheep, goats, buffaloes and camels) are meant to be vaccinated twice a year. However, the analysis of the vaccination data evidences a great heterogeneity in the level of vaccine coverage among species and governorates. Despite the increase in the number of vaccine doses administered in 2015 as compared to 2014, the level of vaccine coverage (30% in sheep and goats, 35% in buffaloes and 60% in cattle) would not be enough to prevent the transmission in the event of RVFV introduction into the country, in particular in those governorates where the level of vaccination is below average. Differences in the density of susceptible species throughout the country combined with the heterogenous application of vaccination programs among areas and species results in the identification of some areas of high density of susceptible hosts of RVFV, which is where efforts should be focused. Failures in the local application of vaccination programs were identified as one of the causes of previous epidemics of RVFV in Egypt [6]. There were also areas where high levels of vaccine coverage were reached resulting in a significant reduction of the risk of RVFV transmission. Conclusions in relation to vaccine coverages should be taken with caution, as the censuses used for their calculation may not be totally accurate/updated.

Even though we used the distribution of *Cx*. *pipiens* to map vector distribution, we believe it is an accurate representation of the RVFV transmission by vectors. Entomological surveys indicate that *Cx*. *pipiens* is the most common mosquito species in Egypt [19,47] and was considered the primary vector of RVFV in previous epidemics [5,16]. Besides, the primary breeding areas of other RVFV vectors (e.g. *Cx*. *antennatus*) coincide with those of *Cx*. *pipiens* [16].

The map of RVFV transmission by vectors in Egypt evidences that the majority of the country surface has a risk of zero, as it corresponds to bare soil land cover (i.e. desert), which is not suitable for the presence of neither hosts nor vectors. Therefore, RVFV transmission by vectors is basically restricted to the wet areas surrounding the Nile River and its delta. Within that area there is a great variability on the risk depending on the density of vectors and hosts. Transmission via mosquitos is considered the most important mode of transmission during the enzootic cycle [2].

The evaluation of the seasonality of *Culex pipiens* indicated that in general there was not much variation in *Cx*. *pipiens* favorability between the different months. Therefore the risk of RVFV transmission by vectors in the suitable areas of Egypt seem to be more or less constant throughout the year. However, results also indicate that in some specific locations there may be significant variations in *Cx*. *pipiens* density. Zayed and collaborators [17] demonstrated that in the areas where the seasonal flooding of the Nile Delta occurred, the population of vectors changed significantly, mainly in late summer-early fall.

The validation of the risk map evidenced that the risk of RVFV transmission by vectors obtained at the location of human and animal cases and RVFV-positive vectors from the previous RVFV epidemics in Egypt, varied between 0.18 and 0.85, with a mean value of 0.49. That would indicate that RVFV transmission by vectors is not restricted only to the areas with the highest risks, but that transmission may occur also in areas where the risks estimated were moderate or even low. In other words, in Egypt RVFV transmission would be possible in areas where the density of vectors or susceptible hosts is far from their maximum values. However, the limitations of the model should also be taken into account. Model results may be influenced by the accuracy of some model inputs (e.g. vaccine coverage), or the uncertainty about some model parameters (e.g. susceptibility of the different species). Besides, knowledge-driven models are always subject to some sort of subjectivity, for example in relation to the factors (layers) chosen. On the other hand, the location of outbreaks used for model validation was not exact, and given that some of them were as old as 1979, the landscape, and therefore the suitability for *Cx*. *pipiens* may have changed since then.

As expected, the linear regression analysis indicated that the distribution of *Cx*. *pipiens* was the most determinant factor in the risk of RVFV transmission by vectors. The only other statistically significant parameter was the density of susceptible goats. Relevance of goats in the risk of RVFV transmission is probably linked to their higher susceptibility, and therefore higher weight, as compared to other species (cattle, buffaloes and camels), as well as with the fact that the areas with the highest density of goats coincided with very low levels of vaccine coverage (7% and 14%, respectively).

The risk of RVFV transmission by vectors in the surroundings of the five quarantines available in Egypt was estimated to be quite low except in the case of Al Qata (where some of the camels imported from Ethiopia arrive). There is no data on the duration of viraemia in camels, but as camels imported from Sudan stay in the quarantine for only 3 days, it seems possible that they may remain infectious after being released from the quarantine, even if RVFV transmission by vectors within the quarantine was not possible. On the other hand, RVFV transmission within those quarantines may also occur by direct contact with tissues, body fluids or fomites of infected animals.

While all cattle are slaughtered within quarantine facilities, imported camels (potentially infected) may be taken to animal markets or to slaughterhouses after the quarantine period, and those would be the places where RVFV transmission would be more likely. The risk of RVFV transmission by vectors at the 273 animal markets was rather variable (between 0 and 0.84), but the mean risk was quite high, 0.60. In the case of the 18 camel slaughterhouses, the risk of RVFV transmission by vectors was quite high (mean risk of 0.59–0.60), and there was in fact less variability than in the case of animal markets. Establishment of an effective surveillance system in the areas surrounding key markets and camel slaughterhouses may allow the early detection of RVF and the timely establishment of control measures. That may include improving passive surveillance by raising farmers’ awareness for the reporting of any symptom compatible with RVF and providing the mechanisms that allowed a prompt laboratory confirmation of any suspected case. Also, the establishment of some active (sentinel) surveillance in the locations identified as having the highest risk would be essential. Besides the risk of RVFV transmission by vectors, the slaughtering of animals, which in the case of cattle is carried out within quarantine premises, poses an important risk for humans by exposure to body fluids or tissues of infected animals. Infectivity of blood during the acute phase of infection in mammals is high [2]. Moreover, given that RVFV is quite resistant to inactivation, infected tissues may remain infectious for a period of a few days [2]. In fact, Nicholas and collaborators [48] concluded that practices such as skinning or slaughtering animals were significantly associated with the risk of RVFV infection. A cross-sectional survey on 1181 abattoir workers in 15 governorates of Egypt after the 1993 RVFV epidemic evidenced a 2% prevalence of anti-RVF IgM antibodies [49]. Antibodies were found in 9 of 31 slaughterhouses and 8 of 15 governorates, including several areas where no clinical disease had been reported. Therefore, Egyptian abattoirs in which imported cattle or camels are slaughtered should implement measures (e.g. use of personal protective equipment) to prevent the infection of the personnel.

Even though we focused on the risk posed by the legal trade of animals, other mechanisms for RVFV introduction into Egypt are also possible. Illegal trade of animals through the borders is likely to occur, but its relevance in relation to the risk of RVFV introduction is difficult to assess, and would require further studies. On the other hand, the possibility of RVFV spread (potentially to distant areas) through the movement of infected (viraemic) humans and subsequent infection of competent vectors remains controversial. While for some authors humans infected by RVFV may develop a viremia sufficient to infect naïve mosquitoes [50], others consider humans as dead-end hosts [3]. Also, wind-borne transportation of infected mosquitos has been proposed [5], but not demonstrated scientifically.
